# “Your whole life is turned upside down”: a reflexive thematic analysis identifying recommendations for designing and conducting qualitative research with cancer caregivers

**DOI:** 10.1007/s00520-025-09493-8

**Published:** 2025-05-06

**Authors:** Katelyn E. Collins, Fiona Crawford-Williams, Chris Sibthorpe, Belinda C. Goodwin, Elizabeth A. Johnston

**Affiliations:** 1 School of Psychology and Wellbeing, University of Southern Queensland, Toowoomba, Springfield, Queensland Australia; 2 Viertel Cancer Research Centre, Cancer Council Queensland, Fortitude Valley, QLD Australia; 3 College of Nursing and Health Sciences, Flinders University, Bedford Park, South Australia Australia; 4 McGrath Foundation, Sydney, New South Wales Australia; 5 Centre for Health Research, University of Southern Queensland, Springfield, Queensland Australia; 6 School of Population and Global Health, University of Melbourne, Melbourne, Victoria Australia; 7 School of Exercise and Nutrition Sciences, Queensland University of Technology, Kelvin Grove, Queensland Australia; 8 Population Health Program, QIMR Berghofer Medical Research Institute, Herston, Queensland Australia

**Keywords:** Cancer survivor, Interview, Person-centered care, Psycho-oncology, Qualitative methodology, Reflexivity

## Abstract

**Purpose:**

There is an increasing need to improve access to person-centered support for cancer caregivers. Qualitative research methods can gather rich insights into the lived experiences of cancer caregivers, providing important information about their unique needs, preferences, and experiences in accessing support for themselves.

**Methods:**

This secondary analysis of a qualitative interview study with cancer caregivers aimed to understand how the design and conduct of interviews can be optimized to center caregivers’ voices and lived experiences. Data from 20 semi-structured interviews with cancer caregivers were analyzed using reflexive thematic analysis to explore underlying patterns in how the interviews were conducted and how caregivers shared their needs and experiences.

**Results:**

Three themes were developed: (1) cancer caregivers’ health and wellbeing was inherently linked to the survivor’s health and wellbeing, (2) question wording and design can perpetuate the “patient focus” that cancer caregivers experience in other settings, and (3) participating in qualitative research can be a meaningful experience for caregivers and provide an avenue to connect them with community-based support.

**Conclusions:**

Researchers should include interview questions which explore dyadic interactions between caregivers and survivors, as well as caregivers’ individual experiences. Practicing reflexivity can increase researchers’ awareness of implicit biases that inform the design and conduct of their research, such as questions that inadvertently shift the focus to the patient rather than the caregiver. Finally, with deeply personal information often disclosed during qualitative interviews, researchers should be equipped to respond with empathy and connect caregivers to professional support as needed.

**Supplementary Information:**

The online version contains supplementary material available at 10.1007/s00520-025-09493-8.

## Introduction

Family and friends play a critical role in cancer care and survivorship [1–3]. As informal caregivers (hereafter, caregivers), their support can reduce the physical, practical, and psychosocial burdens of a cancer diagnosis, resulting in improved mental and physical health outcomes for cancer survivors [4]. Despite their vital contributions, caregivers often report feeling unsupported themselves [5]. Common concerns include feeling underprepared for their involvement in their loved ones’ medical care [6], social isolation from other family members and friends [7], and feeling overlooked by healthcare providers who prioritize the needs of the person with cancer [8, 9]. Thus, there is a critical need to ensure caregivers’ own health and wellbeing is not compromised through their caregiving role [10].

To better understand caregivers’ experiences maintaining their own health and wellbeing, members of the authorship team conducted a qualitative interview study with caregivers living in rural Queensland, Australia. As per the primary research aims, two separate analyses have been undertaken using content analysis to (i) identify changes in caregivers’ health behaviors since caring for someone with cancer [7] and (ii) describe caregivers’ support seeking for their own health and wellbeing [8]. Throughout data collection and analysis, several latent concepts were identified in relation to how caregivers responded to the interview questions which provided further insight into caregivers’ experiences. These concepts extended beyond the aims of the primary analyses and could not be adequately explored using semantic-based content analysis, warranting further investigation via reflexive thematic analysis [11].

This paper therefore reports on a secondary analysis of qualitative data from interviews with cancer caregivers. Reflexive thematic analysis was used to explore underlying patterns in how the interviews were carried out and how caregivers shared their experiences. Findings provide insights into how researchers can design and conduct qualitative research to better understand and support the needs of cancer caregivers.

## Methods

Participant recruitment and data collection methods have been previously reported [7, 8]. In summary, four members of the authorship team (EJ, KC, BG, CS) contributed to the design, conduct, and analysis of semi-structured interviews with cancer caregivers living in rural Queensland, Australia. Ethical approval for this project was obtained from the University of Southern Queensland Human Research Ethics Committee (H17REA152), and all participants provided informed consent (written and/or verbal) prior to participation.

Using a convenience sampling approach, caregivers were recruited through various avenues, including Cancer Council Queensland’s subsidized accommodation lodges, a longitudinal study being conducted by the research team, advertisement in a Facebook support group, and word of mouth. The sample consisted of 20 caregivers, aged 31 to 71 years, half of whom were male, and most (*n* = 13; 65%) being the intimate partner of the survivor. Others were the siblings (*n* = 2), parents (*n* = 2), adult children (*n* = 2), or neighbors (*n* = 1) of the survivor. Caregivers were supporting survivors of various cancers, most commonly head and neck, gynecological, skin, breast, and gastrointestinal. Time since diagnosis ranged from 2 months to 10 years (median 3 years). All caregivers lived in an inner or outer regional area based on the Australian Statistical Geography Standard Remoteness Structure [12].

All interviews were conducted via telephone with the senior author (EJ), a female cancer survivorship researcher (PhD) and Accredited Practicing Dietitian. The interviewer had no prior relationship with any of the participants and had prior training in identifying and managing distress among people affected by cancer. Interviews were audio recorded and transcribed using Panopto [13], with transcripts reviewed by the first author for accuracy (KC). The average interview length was 30 min (range 12 to 52 min).

Primary analysis of the interview data was led by the first author (KC), a female heath psychology researcher (BPsych(*Hons)*), with support from the senior author (EJ) and input from the entire research team. The original analyses utilized content analysis [14] to identify and describe rural caregivers’ changes in health behaviors since supporting their loved one with cancer and their experiences seeking and accessing support for their own health and wellbeing and have been previously published [7, 8]. Throughout data collection and analysis, the research team practiced reflexivity by regularly discussing and noting their assumptions, biases, and perspectives, and how these influenced the construction of meaning from the data [15].

In practicing reflexivity, the research team identified several latent themes that appeared to sit “beneath the surface” of caregivers’ narratives and could not be adequately captured using content analysis [11]. Firstly, it was identified that even when caregivers were explicitly asked about their *own* health and wellbeing, they often spoke on behalf of their loved one. Secondly, cancer caregivers were highly engaged in the research interviews and expressed appreciation for the opportunity to participate. These observations provided the basis for the current study, a secondary analysis of the data using reflexive thematic analysis [16] to explore these latent concepts and the interplay between the design and conduct of the interview, the assumptions of the research team, and how caregivers shared their experiences.

Reflexive thematic analysis is an approach to thematic analysis that acknowledges researchers’ subjective experiences and assumptions in the interpretation of research data [16]. Interview transcripts containing examples of caregivers speaking on behalf of their loved ones, and expressing appreciation for participating in the interview, were reviewed and coded by the first author (KC). Themes were developed in collaboration with the senior author (EJ) and then reviewed and refined with the broader research team, consisting of individuals involved in the primary analyses (BG, CS) and a cancer survivorship researcher with expertise in qualitative methodology (FCW). The analysis is reported according to the Standards for Reporting Qualitative Research guidelines [17] (see Supplementary File 1).

## Results

Three core themes, representing new insights for designing and conducting qualitative research with cancer caregivers, were developed and are presented below.

### Theme 1: we and our—cancer caregivers’ health and wellbeing was inherently linked to the survivors’ health and wellbeing

To contextualize the interview questions, each interview started with the following preface, which outlined the key purpose of the study and the focus on caregivers’ experiences: “We are looking at ways we can support the health and wellbeing of people who are living in rural areas and supporting someone with cancer.” Interview questions were designed to engage caregivers in discussions about their needs and experiences seeking support for themselves, and caregivers were explicitly queried about changes to their own health behaviors since caring for a loved one with cancer. Throughout the interviews, however, caregivers frequently responded to questions about *their* experiences with information about the *survivor.* In many instances, the interviewer had to clarify the focus of the original question. For example:

#### Interview 15

Interviewer (I): *“Have your sleep habits changed since the diagnosis?”*

Caregiver (C): *“Yeah, because my wife finds it very difficult to sleep in the bed, so she basically sleeps in a comfortable, nice lounge chair…”*

I: *“And what about* your *sleep? Have* you* noticed any changes?”*

C: *“Well, I did at one point, but I don’t think it’s because of taking care of her. I think it was because of my own mental health problems.”*

#### Interview 18

I: *“Did your diet change after the diagnosis happened?”*

C: *“Oh, when my husband first started treatment, he didn’t feel like eating – because you know, when they go through radiation and all that.”*

I: “*Yeah, and how was* your *diet? Did* you *notice any changes?”*

C: “*Oh, when you’re away from home, you always eat different. Yeah, we ate differently.”*

In some instances, caregivers described changes in their health behaviors as something experienced collectively by themselves and their loved one, using language such as “we” and “our.” This was especially common among caregivers supporting their intimate partners.

#### Interview 19

I: *“Did you notice any changes to how active you were with the extra caring work?”*

C: *“Yeah, I probably – we weren’t doing the normal amount that we would, like going to the shops, or going to the hardware store on Sundays. We did a few projects and things that kept my wife’s interest while she was at home.”*

#### Interview 20

I: *“[Did you notice any changes in] your alcohol consumption?”*

C: *“Our consumption didn’t really increase during my wife’s treatment.”*

Initially, we assumed this phenomenon was a misunderstanding of our research aims. However, even when caregivers were explicitly reminded of the study’s focus (i.e., their own health and wellbeing), they continued to discuss the survivor and their shared experiences:

#### Interview 13

I: *“The next few questions are just about your own health and wellbeing… What about physical activity? Are you as active, or more active [than you were]?”*

C: *“We’re not more active. A lot less active than what we were… My wife doesn’t like the idea of being on a bike and feeling the seat, so we don’t do that much.”*

Reflecting on our professional backgrounds as health researchers, we acknowledge that at times we can view health behaviors and support seeking through an individualistic lens. However, this theme highlighted that caregivers’ health and wellbeing may be closely intertwined with that of the survivor, hence caregivers’ recollections of shared experiences and changes. Similarly, caregivers also described using coping strategies that dually impacted themselves and the survivor, such as refraining from sharing their concerns or emotions with the survivor to prevent causing them distress.

#### Interview 1

C: *“Your whole life is turned upside down... The biggest challenge is to try and appear that you’re not in any way affected, ‘cause that would cause stress to the person you’re caring for.”*

Minimization of caregivers’ own needs and experiences was furthered by comparing their situation to the cancer survivor.

#### Interview 3

C: *“I just kept thinking, oh, I’m doing fine, no problem… but after [supporting my sister in hospital] for ten days yesterday, I felt guilty. I thought, yes, it is affecting me*. *I’m by myself. But you know, my experience is nothing in comparison to what my sister has been through.”*

#### Interview 11

C: *“A lot of people ask me, what about you? Yeah, I’m fine. It just takes it out of you, but overall, I’m a lot better off than my wife. She would do it for me, so why can’t I do it back? If you really love someone, you make sure they’re as comfortable as they can be.”*

Some caregivers appeared to gather strength from, and even reported feeling “supported by,” their loved one’s resilience in coping with cancer.

#### Interview 6

I: *“Do you have any final comments or thoughts about looking after your own health and wellbeing while supporting your partner?”*

C: *“… I’ve been amazed at my partner’s inner strength. She’s not the type of person to lay in bed all day feeling sorry for herself. When she’s having chemo, the first few days knock her about a bit… but after those days wore off and she started to improve, she’s up and about.”*

These findings highlight the interrelationship between caregivers’ and survivors’ health and wellbeing and the role that their relationship dynamics can play in coping with cancer, including changes in caregivers’ health behaviors and support seeking. Therefore, explicitly asking caregivers about the impact of their loved ones’ needs, preferences, and behaviors on their own health behaviors and ability to seek health and wellbeing support may provide further insights into caregivers’ experiences.

### Theme 2: question wording and design can perpetuate the “patient focus” that cancer caregivers experience in other settings

*Theme 1* explored how caregivers discussing the survivors’ experiences when asked about their own may demonstrate an interrelationship between caregivers’ and survivors’ health and wellbeing. However, knowing that data are co-produced in qualitative research interviews [18, 19], we also reflected on the choices we made, as researchers, in designing and conducting the interviews to provide additional insights into this phenomenon.

Although the aim of the interviews was to center the experiences of caregivers, the very first question we asked was, “Could you tell me a little bit about the person you’re supporting?”. This question was intended to enable description of the study sample and contextualize caregivers’ experiences. However, in reflecting on the implicit power that researchers can hold as “professionals” or “knowers” [20], it is possible that prefacing the interviews with this question may have perpetuated the same “patient focus” that caregivers report experiencing in other professional and social settings. Indeed, this experience was highlighted by caregivers in our interviews, who felt their needs were, at times, not acknowledged or valued by the patient’s healthcare professionals or their acquaintances, as the caregiver was not the one diagnosed with cancer [8].

We do not have any reason to believe that the design of our interview caused harm or distress for any interview participants, as many were highly engaged during the interview and appreciated the opportunity to share their personal experiences (see *Theme 3*). Rather, we hypothesize that opening the interview with a question about the patient, rather than the caregiver, implied that the survivor’s experiences were a key focus of the research. Although we were not able to test this hypothesis, this finding highlights the importance of practicing reflexivity. Reflecting on our assumptions, biases, and decisions as researchers provided insights into how we could further center the voices of caregivers in our interviews. For example, interviews could begin by asking caregivers to describe a typical day for them in the past month. Additionally, questions about the patient’s diagnosis and treatment status could be addressed at the end of the interview (if not already answered), to maintain focus on the caregiver.

### Theme 3: participating in qualitative research can be a meaningful experience for caregivers and provide an avenue to connect them with community-based support

The final theme illustrates the apparent meaning that caregivers derived from participating in qualitative interviews. While conducting the interviews, we were mindful of participant burden, a key principle of ethical human research [21]. Accordingly, we aimed to complete the interviews within 20 min to minimize participant burden. However, we found that caregivers were highly engaged and shared openly about their experiences, with interviews averaging 30 min in length, and some extending beyond 40 min. Our initial concern that a long, in-depth interview may be burdensome to caregivers was not reflected by participants. At the end of the interview, many caregivers expressed appreciation for the opportunity to take part and for the research team’s interest in understanding their experiences and needs:

#### Interview 3

I: *“Is there anything else you wanted to mention? That’s all the questions we had.”*

C: *“No, I think that’s fine. Thank you for suggesting the phone number for counselling… Look, I’ll let you go. Thank you very much for your interest and for this project, and what you’re doing.”*

#### Interview 5

I: *“Thank you so much for all the valuable insights you’ve shared today. That’s been really helpful. Did you have anything else to say?”*

C: *“… I guess, just, thank you… I appreciate the opportunity as well, so thank you.”*

Given that caregivers’ expressions of appreciation typically occurred upon interview close, adherence to social norms of providing thanks at the end of an interaction may account for at least some of this phenomenon. However, it is notable that caregivers typically expressed their thanks in response to the interviewer providing them with a final opportunity to share further details about their experience maintaining their health and wellbeing and seeking support that they had not yet had the chance to share in the interview. As such, it appears that some caregivers genuinely perceived the interviews as a positive and rewarding experience. Another caregiver even described participating in the research interview as a supportive experience while navigating their loved one’s cancer diagnosis.

#### Interview 15

I: *“Has there been any particular support services or people that have been really helpful in this time?”*

C: *“…. The research you’re doing has been helpful in the sense that it’s kept us aware and increased our awareness of the difficulties of cancer.*”

Cancer caregivers may therefore derive support and meaning from qualitative research participation beyond what is typically appreciated by researchers. Prior research indicates that caregivers can experience several barriers to accessing support for their own health and wellbeing, including limited knowledge of available services and reduced capacity to navigate support services alone [8]. While the support provided by researchers during interviews cannot replace the support provided in a clinical, therapeutic context, research interviews can present an opportunity to provide catharsis and connection for caregivers through the opportunity to share personal experiences with an attentive listener (i.e., the researcher). Similarly, the interviews were also an opportunity to provide caregivers with information about professional community-based support, particularly when they expressed a need for support beyond the researcher’s scope of practice. As shown below, caregivers often indicated that they were unaware of the support services offered and were grateful for the recommendation.

#### Interview 15

C: *“I truly believe that if a support service could provide access to a psychologist for the person that is taking care of the patient, that would be very useful.”*

I: *“… If you did want to reach out,* [community cancer support service] *has a phone line, and you can access psychology and counselling through them.”*

C: *“I’m going to write that down.”*

In summary, research interviews can be a supportive environment for caregivers to share their personal experiences and to receive information about professional community-based supports where appropriate—both of which are within researchers’ scope of practice to provide.

## Discussion

**Fig. 1:**
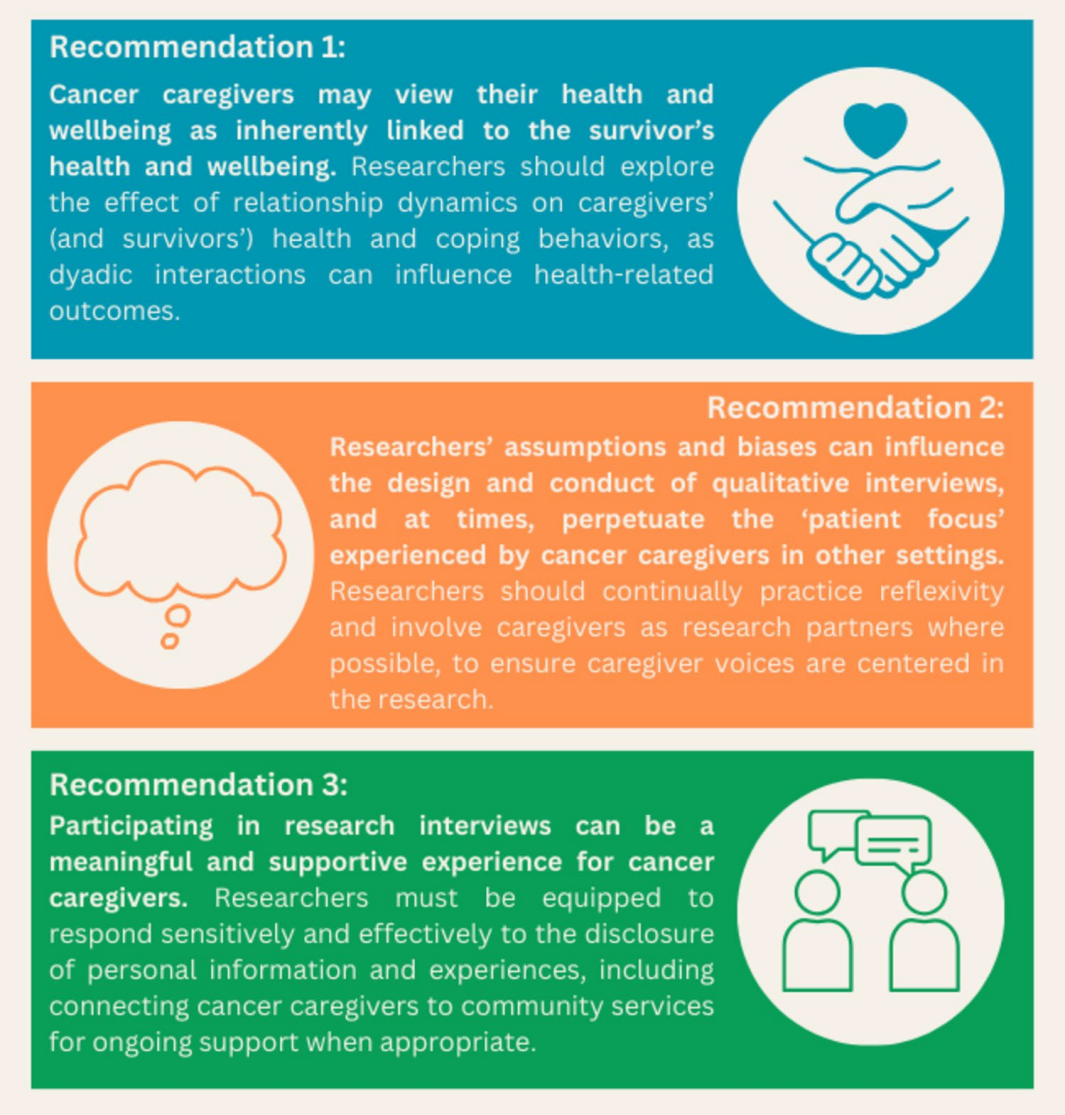
Summary of key recommendations for designing and conducting qualitative interviews with cancer caregivers

Using insights gained from reflexive thematic analysis, this paper provides practical recommendations for designing and conducting qualitative interviews with cancer caregivers, ensuring their voices and experiences are centered (see Figure 1).

Firstly, our findings indicate that caregivers may view their health and wellbeing as inherently linked to that of the cancer survivor. This aligns with the concept of “dyadic coping” where caregivers and their loved ones (particularly intimate partners) mutually influence one another’s coping and adjustment to stressful life events, such as a cancer diagnosis [22]. These dyadic coping responses can enhance or hinder functioning among members of the dyad. For example, our analysis identified that caregivers may conceal or minimize their distress to protect the survivor, a dyadic coping behavior known as “protective buffering” [23]. While our interviews focused on the experiences of caregivers, research suggests that protective buffering can be bidirectional, with survivors also “buffering” caregivers to reduce caregiver burden and distress [24, 25]. However, this coping strategy is generally linked to poorer relationship satisfaction and mental health for both dyad members [24, 25], potentially because protective buffering replaces open communication and heightens feelings of isolation. Instead, cancer survivor-caregiver dyads who utilize “active engagement,” a stress management approach involving collaborative problem solving [23], report greater relationship satisfaction [26]. While caregivers’ experiences should not be minimized in favor of the survivors’, our findings indicate that a comprehensive understanding of caregivers’ health and wellbeing should consider both individual and dyadic coping behaviors, providing a nuanced perspective on caregivers’ needs and experiences. Further, it is possible that caregivers’ personal characteristics and the nature of their caregiving role may influence how they relay their experiences of caring for someone with cancer. For example, dyadic coping among intimate couples may differ compared to that of parent-child dyads, who may be less likely to view themselves as a “unit.” Future research could consider the impact of caregiver and survivor characteristics on dyadic coping and how this may manifest in research interviews.

Additionally, we propose that the design and conduct of interviews may influence how caregivers share their experiences. Commencing the interviews with a question about the patient, rather than the caregiver, may have detracted from our study aim of centering the experiences of caregivers, influencing their responses in turn. Previous work highlights the importance of directly involving the target population (e.g., caregivers) in the design and conduct of research, ensuring evidence-based solutions are relevant and useful [27]. For example, a recent study reports on ten principles for designing qualitative survey questions that were generated in focus groups and interviews with community members, including a large number of cancer caregivers [28]. These principles, such as avoiding assumptions or leading questions, could be used to design questions for qualitative interviews with caregivers. Further, piloting the interview guide with caregivers and seeking their feedback on its design and conduct can assist researchers with developing an interview that is sensitive and relevant to their needs and preferences.

Finally, we found that caregivers value the opportunity to participate in research and share their personal experiences. In palliative care settings, patients receiving end of life care and their caregivers also report benefits from research participation, such as satisfaction from contributing to scientific knowledge and processing their experiences, even when they are unlikely to directly benefit from the outcomes of the research [29, 30]. Research interviews can therefore provide an acute form of support to caregivers by creating a space for them to “offload” and share their experiences, including information which may not have been openly shared before. Our interviews also found that caregivers are often unaware of where to access support for themselves; thus, researchers should be prepared to direct caregivers to relevant community-based services as needed. Future research could explore effective communication strategies for researchers to use when caregivers require support that is beyond the researcher’s scope of practice, ensuring that qualitative interviews are a supportive experience for both caregivers and researchers.

## Conclusion

This reflexive thematic analysis of semi-structured interviews with cancer caregivers provides practical recommendations for researchers designing and conducting qualitative research with this group. Researchers should consider both the dyadic relationship between cancer caregivers and survivors as well as their own values and assumptions, including how these may perpetuate the “patient focus” that caregivers can experience in other settings. Finally, participating in qualitative research can be a supportive experience for caregivers, enabling them to feel valued and understood. Researchers should be equipped to respond to caregivers’ narratives with sensitivity and to provide information about community-based support services when appropriate.

## Supplementary Information

Below is the link to the electronic supplementary material.

Supplementary file1 (DOCX 28 KB)

## Data Availability

The data are not publicly available due to privacy or ethical restrictions.
